# Investigation into the rockfall impact process of a quarry landfill slope under highway expansion

**DOI:** 10.1007/s11069-024-06980-9

**Published:** 2024-11-20

**Authors:** Bin Gong, Xiang Yu, Yongjun Zhang, Chunyan Bao, Chun’an Tang

**Affiliations:** 1 Department of Civil and Environmental Engineering, Brunel University London, London, UB8 3PH UK; 2 State Key Laboratory of Coastal and Offshore Engineering, Dalian University of Technology, Dalian, 116024 China; 3 School of Civil Engineering, Qingdao University of Technology, Qingdao, 266520 China; 4 College of Civil Engineering, Shaoxing University, Shaoxing, 312000 China

**Keywords:** Rockfall, Slope failure, Quarry landfill slope, Risk assessment, Protection measures

## Abstract

A quarry landfill slope is commonly partially or entirely filled with quarry waste. On the surface, a substantial amount of rough stone waste accumulates. This study specifically investigated the hazards posed by individual rockfalls and cluster rockfalls induced by landslides in such slopes, using an engineering slope as an illustrative example. The discontinuous deformation and displacement analysis method was employed to analyze the individual and cluster rockfall motion characteristics, as well as the dynamic response of protection structures. The results indicate that: (1) The impact of individual falling rocks on structures results in deformation and damage that far surpasses that caused by a flat plane impact. Interestingly, the stress generated upon rockfall contact with the structure is not initially at its maximum; it gradually increases to a peak as deformation occurs. When the structure is damaged or rebounds, the impact stress significantly diminishes. For wedge-shaped falling rocks impacting the upper part of the structure, bending tilting failure tends to occur. Conversely, irregular blocks with larger volumes impacting the lower part of the structure often lead to direct toppling failure; (2) Clusters falling rocks impede the movement of the sliding body. As the front and rear sliding bodies fracture along the middle, the rear sliding body tilts. Consequently, accumulated blocks are struck by the sliding body, initiating oblique throwing movements. There is a high likelihood of these rocks crossing protective structures; (3) The protection rate of the protective structure against single block stone impact stands at 86.7%. However, when subjected to the impact of a group of rockfalls, the protective structure completely fails. Overall, although the current protective measures are relatively cost-effective, the extremely high probability of casualties makes them unacceptable.

## Introduction

Rockfall, a common and serious geological hazard worldwide (Walton et al. 2023; Ma et al. 2021), poses significant risks to lives, infrastructure, and transportation systems due to its high energy and mobility (Guzzetti et al. 2003; Crosta and Agliardi 2020). Consequently, establishing effective protection structures is of paramount importance. Current research on rockfall protection can be broadly categorized into three approaches: The first one employs large pendulums or free-falling weights to impact various protection structures, including sand cushions (Natio et al. 2020; Wang et al. 2022; Shen et al. 2021), geofoam (Zhao et al. 2023), ring net barriers (Liao et al. 2024; Korini et al. 2021), and rock sheds (Ertugrul and Kiwanuka. 2023; Liu and Liao 2022; Shen et al. 2020). These studies analyze the dynamic response characteristics such as impact force, displacement, duration, and stress within the protection structures to evaluate their buffering and energy dissipation performance. The second one treats protection structures as rigid bodies, which the rock blocking rate is evaluated by assessing whether the rockfall trajectory intersects with the protective structure (Kanno et al. 2021; Akin et al. 2021; Singh et al. 2021). Alternatively, the structure size and layout position are determined based on the trajectory of rockfall in the absence of protection (Alemdag et al. 2022; He et al. 2021; Kanno et al. 2023). The research on rockfall trajectories can be further categorized into on-site investigation (Prades-Valls et al. 2022; Chang et al. 2023), physical experiment (Nakajima et al. 2021), block or particle flow simulation by considering the shape of falling rocks (Bourrier et al., 2022; Fan et al. 2022; Wu et al. 2022), and statistical analysis that treats falling rocks as particles and disregards their shapes (Vanani et al. 2024; Farmakis et al. 2022). The third one inputs calculated parameters such as rockfall speed, height, energy and protective structure details into either a neural network (Marchelli et al. 2021) or a semi-probabilistic design framework (Biagi et al. 2020). By conducting sensitivity analyses, the safety factor and reliability of a protection structure are quantified. Notably, all these studies primarily focus on rockfall protection for soil and rock slopes, providing valuable guidance for parameter design and layout of structures.

However, few researches have specifically focused on the risk of rockfall protection on quarry landfill slopes. These slopes are partially or entirely filled with quarry waste soil, and a substantial number of stones rest on their surface. When subjected to slight disturbances, these stones may move downward, resulting in rockfall disasters. Despite their seemingly inert nature, the large volume and density of these stones make them equally hazardous as natural rock blocks. Moreover, the composition of most quarry waste soil consists of gravel and construction waste, rendering it loose and having low bearing capacity (Rahmani et al. 2020; Zhao et al. 2018; Guo et al. 2021). Consequently, arranging protection structures near the rockfall source area becomes unfeasible. Instead, protection structures can only intercept falling rocks at the middle or end of their trajectories. Due to the complex dynamics involved such as collision, rebound, rolling, sliding, and free fall during rock movement (Varnes 1978), blocks after traveling long distances often possess significant kinetic energy (Ji et al. 2023) and unpredictable trajectories (Zhang et al. 2022). Therefore, comprehensive research on rockfall protection necessitates not only calculating the trajectory but also analyzing the dynamic response and failure characteristics of protection structures as they move along the slope. At present, this topic is still challenging.

In addition, loose quarry waste soil is prone to sliding after rainfall because rainfall will weaken soil strength, reduce safety factor and promote the formation of failure surface (Gong 2021; Yu et al. 2021a, 2023, 2024). This instability affects the stones on the surface, leading to cluster rockfall disasters. Since the soil lacks cohesion, the landslide body inevitably experiences relative friction and movement with the blocks. When blocks become unstable, they may also collide or rub against the landslide masses. The mechanism and movement characteristics of this soil-rock binary structure disaster differ significantly from debris flow, where clayey soil encircles blocks and moves downward simultaneously (Martinengo et al. 2021; Tang et al. 2023; Cheng et al. 2023). However, few researchers have yet conducted dynamic analyses on this type of disaster and analyzed the dynamic response of protection structures under disaster impact.

In summary, rockfall hazards on quarry landfill slopes pose great danger and exhibit intricate deformation and failure mechanisms. These hazards have received limited research attention. Based on this, a quarry landfill slope associated with an expressway expansion project is taken as an example in this paper. First, through on-site investigation, geological survey and displacement monitoring, the rockfall source area was precisely located, and rainfall was emerged as the main causes of landfill deformation. Meanwhile, considering the layout and size of protection structures, the discontinuous deformation and displacement (DDD) method was used to analyze the motion characteristics of individual rockfalls and their impact on the structure. Furthermore, this method is combined with the finite element method to study the deformation and movement of the landfill and cluster blocks from stability to instability under rainfall conditions, as well as the dynamic response of protection structure under the disaster impact. The DDD method integrates the rock failure process analysis (RFPA) and discontinuous deformation analysis (DDA) methods and allows to simulate the movement of irregular rock blocks and assess the real dynamic response and damage characteristics of the structure upon impact.

## Overview of slope

### Basic characteristics

**Fig. 1:**
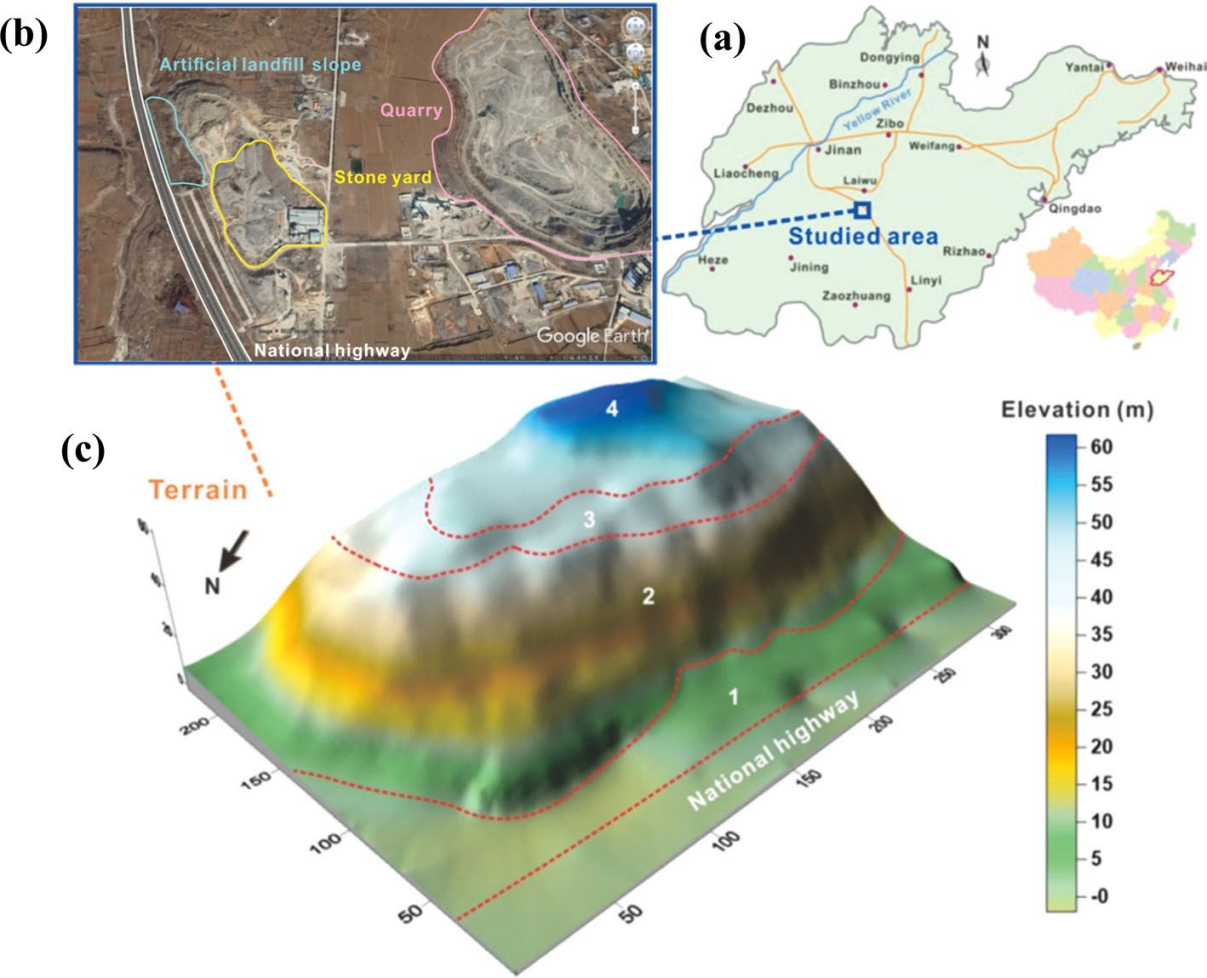
Basic characteristics of the quarry landfill slope: **a** geographical location, **b** surrounding environment, **c** topographic map

The K580 + 265 ~ K580 + 591 slope is located in the southern Laiwu District, Jinan City, Shandong Province, China, as shown in Fig. 1a. Its western side abuts a national highway, while the eastern and southeastern flanks close to an abandoned quarry and a stone yard, respectively, as shown in Fig. 1b. The topography exhibits a pronounced elevation difference, with the southwest being higher than the northeast by a maximum of 58 m. The slope has an inclination of 254° and an average slope angle of 36°. The slope can be divided into four distinct areas from top to bottom: Regions 3 and 4 are second and third level slopes, each with a height of 10 m, filled by gravel and waste from the quarry. Region 2 is the first level natural slope with a height of 35 m. Region 1 serves as an expansion area between the slope toe and the highway, as illustrated in Fig. 1c. Notably, the highway remains operational during the construction process.

### Geological hazard

**Fig. 2:**
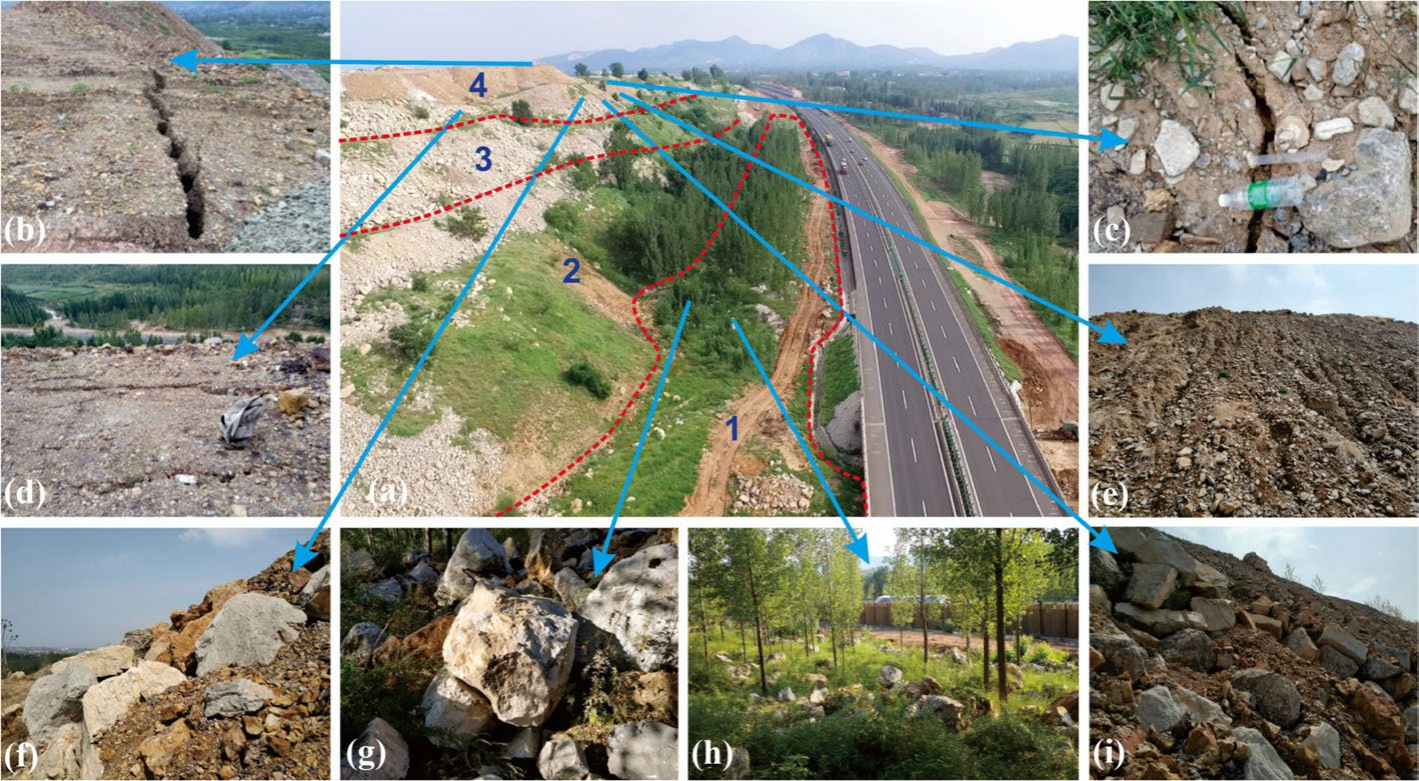
The disaster characteristics: **a** UAV images of slope, **b**, **c**, **d** tension cracks, **e** rain erosion, **f**, **i** accumulation of block stones at the middle of the third level slope, **g**, **h** block stones accumulation at the slope foot and bottom (Note: 1, 2, 3, and 4 in Fig. 2a represent the construction area for slope bottom, the primary natural slope, and the secondary and tertiary accumulation bodies, respectively.)

The planned slope expansion was set to commence in September 2018. In July of the same year, the construction team employed a unmanned aerial vehicle (UVA) to capture slope images. The results showed that the surface of the third slope was covered by talus, the first natural slope and upper first slope was covered by gravel. The middle and lower sections of the first slope, as well as the expansion area, were covered by herbaceous and arboreal vegetation, as shown in Fig. 2a.

The further on-site investigation revealed the critical aspects of the second and third-level landfills. These landfills consist gravel and soil, which exhibit extreme looseness and deformation. Two tension cracks were exposed in the middle of the third level slope top, with the longest length of 40 m and a width of 10 ~ 170 cm, as shown in Fig. 2b. Three cracks were found on the south secondary slope top, with the longest length of 4 m and an average width of 15 cm, as shown in Fig. 2c. Six tension cracks were found on the north side, with an average penetration length of 10 m and a width of 5 ~ 20 cm, as shown in Fig. 2d. Combined with the surface runoff erosion marks observed on the west slope of the secondary landfill shown in Fig. 2e, it suggests that the landfill body may be susceptible to further deformation or even loss of stability under external disturbances.

Apart from the landfill, substantial rubble was observed in the middle of the third-level slope surface, as depicted in Fig. 2f and i. This phenomenon was also evident at the toe and bottom directly below the source area, as illustrated in Fig. 2g and h. By comparing the lithology and shape characteristics of the blocks in these areas, it has been determined that the rubble at the slope bottom results from rockfall. Some of these blocks are in close proximity to the highway.

### Survey and monitoring

**Fig. 3:**
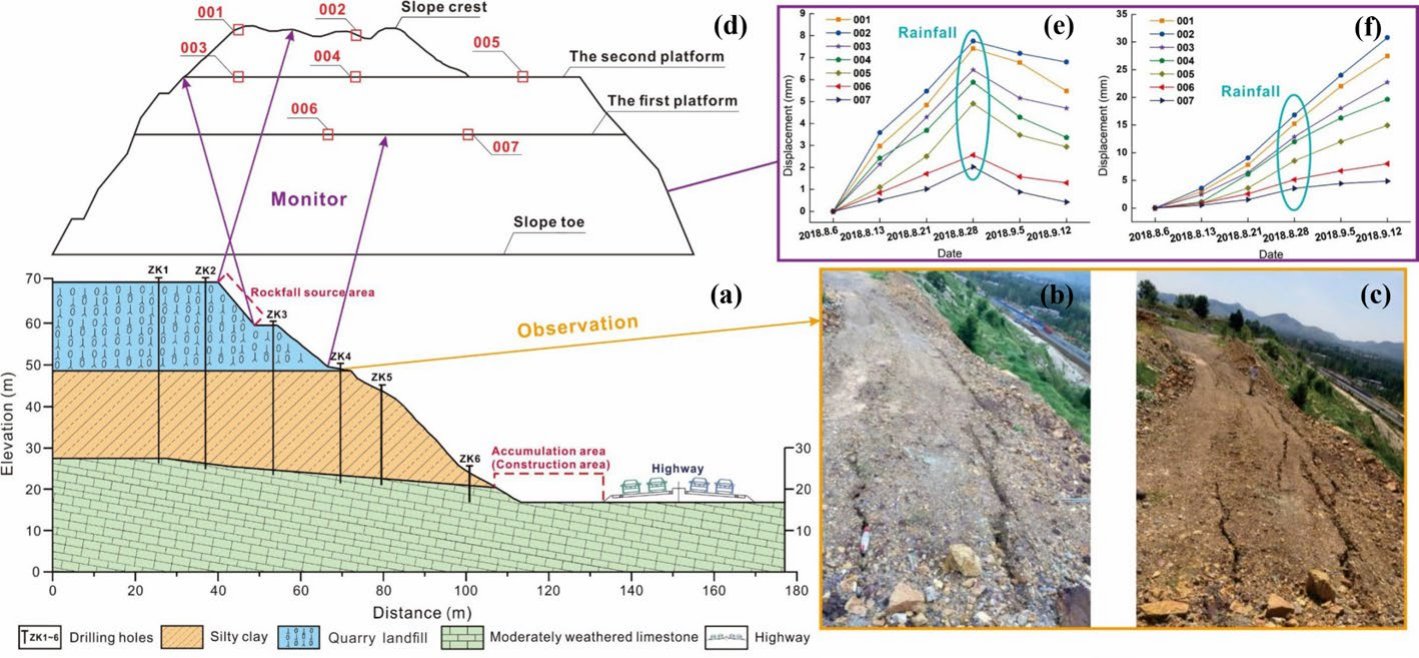
Field investigation and monitoring: **a** geological profile of K580 + 485 section; **b** cracks before and **c** after rainfall; **d** schematic diagram of the measuring point layout; **e** horizontal displacement increment and **f** accumulation

To explore the geological structure of slope body, the construction team conducted six geological drilling holes along the central axis of the rockfall source area. These holes had depths ranging from 11.2 to 43.7 m, as depicted in Fig. 3a. The findings revealed a stratification comprising three layers, from top to bottom: plain fill, silty clay, and moderately weathered limestone. These layers correspond respectively to the landfill body, the natural slope, and the area extending from the slope toe to the bottom. The plain fill primarily consists of limestone gravel, characterized by loose soil that is susceptible to erosion. In contrast, the silty clay soil is slightly denser than the former, with a yellow–brown core displaying blue-gray color. The weathered limestone core remains intact, exhibiting a down-dipping layered structural rock formation—an unfavorable condition for slope stability. No fault structures or active fault zones were identified at the site. Groundwater originates from bedrock fissures, and stable groundwater levels have not been observed during exploration.

Furthermore, the slope monitoring commenced on August 6, 2018, following a weekly cycle to track the horizontal displacement of the slope crest and platforms, as illustrated in Fig. 3b. During the period from August 23 to 26, the cumulative rainfall on the slope amounted to 175.5 mm. Subsequently, the horizontal displacement of the slope exhibited a pronounced increase, as depicted in Fig. 3e and f. Following an on-site survey conducted on August 28, two fresh cracks emerged on the southern secondary slope, measuring an average length of 10.5 m with a maximum opening width of approximately 270 mm. Additionally, the maximum opening width of the pre-existing through-cracks expanded to 20 mm. These observed signs suggest that the landfill may continue to deform or even become unstable during rainy conditions.

Based on geological surveys and monitoring, it has been observed that the landfill body is loose, with a significant accumulation of rubble on its surface. The rubble is prone to downward movement, potentially causing individual rockfall incidents. Additionally, during rainfall, the landfill may slip, leading to instability of the whole rubble. This instability could result in a cluster of rockfall hazards, posing risks to both construction workers and highway traffic. To ensure safety in the construction area and along the highway, the installation of rockfall protection structures is essential.

### Existing rockfall protection

**Fig. 4:**
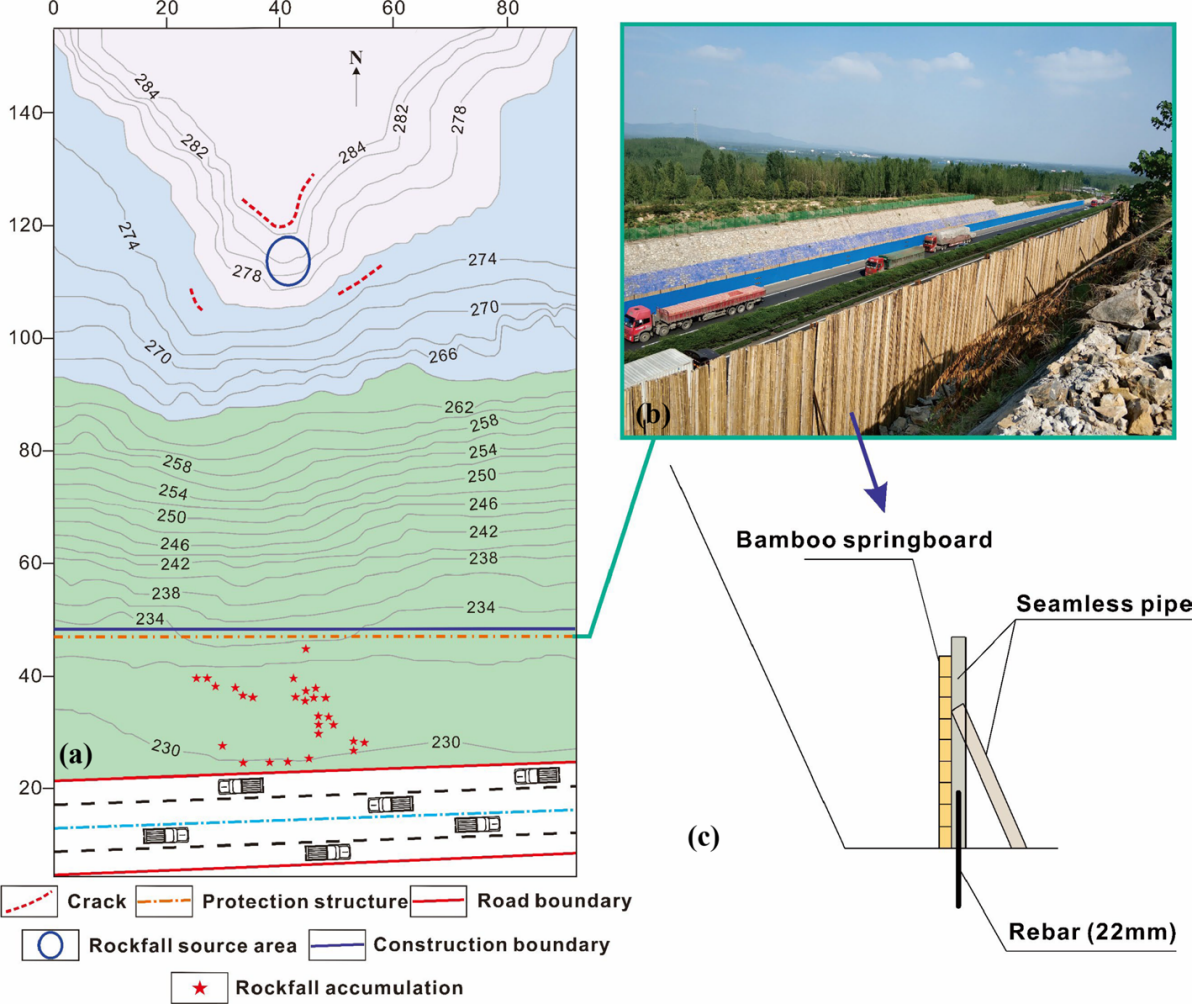
Layout of rockfall protection structure: **a** topographic plan of slope area, **b** protection structure on site, and **c** composition of protection structure

In September 2018, prior to the commencement of highway expansion, the construction team opted for a temporary protection structure of 3 m × 0.5 m. This structure was composed of bamboo springboards, seamless steel pipes, and 22-mm threaded steel bars, designed to intercept falling rocks, as shown in Fig. 4c. Given that the source area lies on the third-level slope, and the protection structure must be installed on a flat surface, the most suitable location for deployment would be the second or first-level platform. This strategic placement ensures that falling rocks can be intercepted during the early or intermediate stages of their movement. However, due to the extremely loose composition, the landfill lacks sufficient foundation bearing capacity and frictional resistance to support the structure effectively. Consequently, the structures has to be positioned at the slope toe, minimizing any impact on ongoing construction activities, as shown in Fig. 4a and b.

As a result, the protection structure can only intercept rockfall at the end of its movement. During their descent, falling rocks collide, rebound, roll, slide, and fall freely. Consequently, there is a possibility that they might pass over the structure and enter the construction area. Simultaneously, the rubble moving toward the slope toe possesses significant kinetic energy. The impact from individual and clustered rockfalls could potentially overturn or damage the structure, rendering it ineffective and jeopardizing the safety of both the construction site and the highway area. In light of this, the subsequent chapters will conduct a risk analysis of individual and clustered rockfall incidents, evaluating the interception effectiveness and the impact of rubble on the protective structure.

## Analysis of individual rockfall

### Statistical survey on rubble

**Fig. 5:**
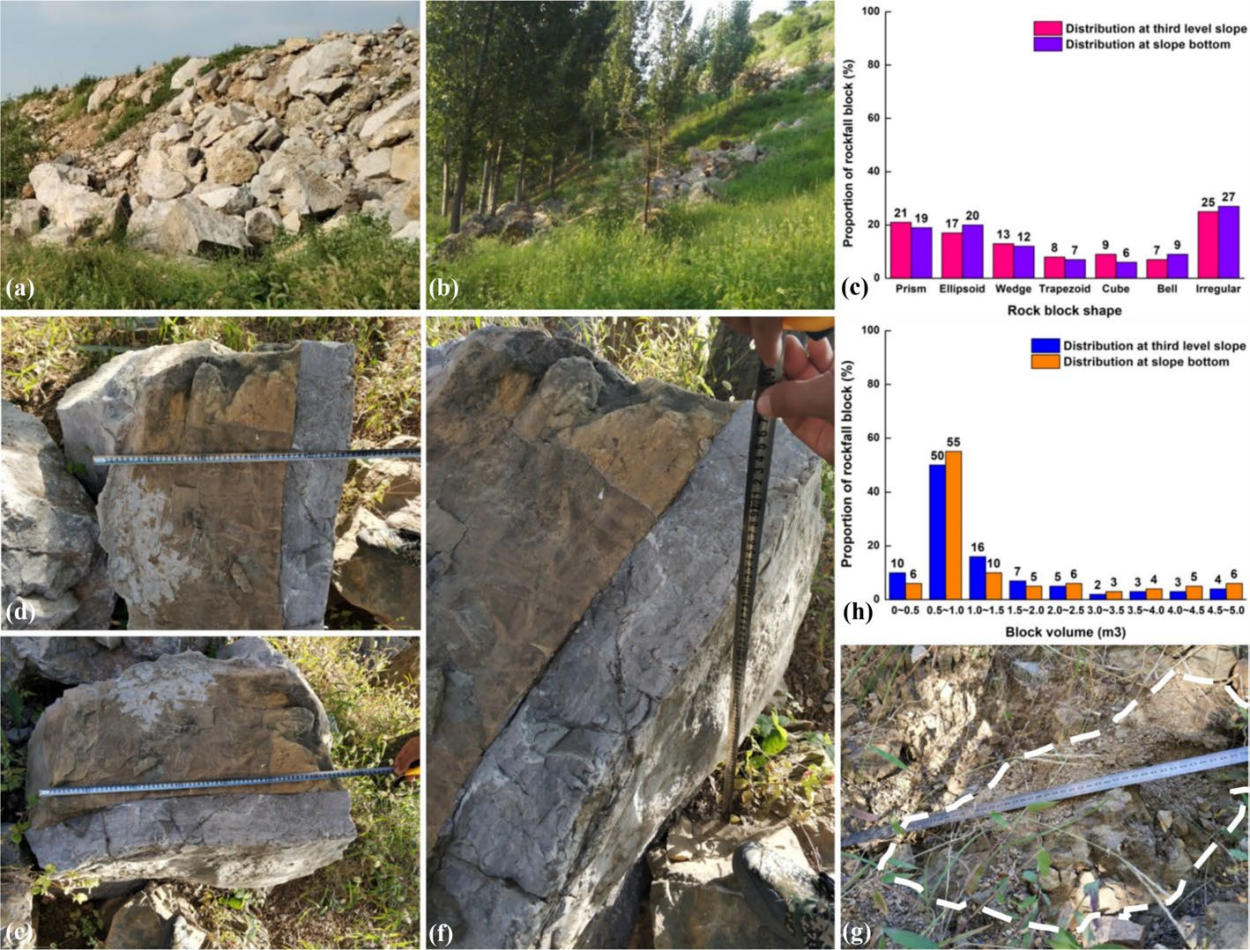
Statistical investigation of rubble: **a**, **b** accumulation of rubble at the third-level and slope toe, **d**, **e**, **f**, **g** measurement of rubble size, **c**, **h** distribution of rubble size and shape

Statistical investigations reveal that the rubble consists of moderately weathered limestone, characterized by irregular shapes and haphazard accumulation. Stacking phenomena occur both at the slope bottom and within the source area, as depicted in Fig. 5a and b. On average, a single section of the rockfall source area contains 15 pieces of rubble. These fragments exhibit diverse forms, including prisms, ellipsoids, wedges, trapezoids, cubes, bell shapes, and other irregular configurations. Among these, irregular blocks constitute the highest proportion, accounting for 25% and 27% of the total number of third-level slopes and slope bottoms, respectively. Following closely are prisms and ellipsoids, which both make up approximately one-fifth of the total count in the two areas. The wedge-shaped bodies rank fourth, representing 13% and 12% of the total count in the respective regions. The distribution of other block shapes is relatively uniform, as depicted in Fig. 5c. The measured particle size range for these blocks spans from 68.73 cm to 128.72 cm, with corresponding weights ranging from 5.07 kN to 122.62 kN, as illustrated in Fig. 5d to g. The size of rubble greatly varies from 0 ~ 5.0 m^3^. Notably, the blocks with a volume of 0.5 to 1.0 m^3^ account for more than 50% of the total.

### Basic principles of the DDD method

Research indicates that the trajectory is influenced by both the shape and quality of the rock blocks. Given the significant variation in the shape and size of rubble, it becomes essential to characterize these factors through numerical simulations. In this study, the rockfall source and accumulation area are situated near the K580 + 485 section, as depicted in Fig. 4a. To analyze the risk associated with individual and cluster rockfall events, this section is chosen to build a two-dimensional model, and the DDD numerical method proposed by Gong et al. (2018) and Gong et al. (2019a) is adopted. By combining the RFPA (Tang 1997; Chen et al. 2022; Wang et al. 2023) and DDA methods (Shi et al. 1985; Gao et al. 2024), a comprehensive simulation that considers both microscopic material damage and large block displacement is achieved. Notably, this method surpasses the DDA approach by accounting for unit non-uniformity and simulating stress–strain behavior within a single element (Gong et al. 2019b), as elaborated in the subsequent sections.

#### (1) Displacement function and equilibrium equation

In the DDD method, triangular elements are used to construct numerical models. The displacement function of each element bears resemblance to the shape function used in finite element models. Specifically, the displacement fields are computed based on the displacements of the element nodes. For a three-node triangular element denoted as *m*, which possesses six basic degrees of freedom, the displacement components of an element can be expressed as follows:

1 $$\begin{array}{*{20}c} {\left[ {D_{m} } \right]^{T} = \left[ {\begin{array}{*{20}c} {u_{i} } & {v_{i} } & {\begin{array}{*{20}c} {u_{j} } & {v_{j} } & {\begin{array}{*{20}c} {u_{k} } & {v_{k} } \\ \end{array} } \\ \end{array} } \\ \end{array} } \right]} & {\left( {m = \, 1, \, 2, \ldots ,n} \right)} \\ \end{array}$$

where *u* and *v* are the two displacement components of a node along the *x-* and *y-*axis, respectively; *i*, *j* and *k* are the three nodes associated with the element *m*;* n* is the total number of all elements*.* For any point (*x*, *y*) located inside the element *m*, the displacement vector [*u v*]^T^ can be expressed as follows:

2 $$\begin{array}{*{20}c} {\left[ {\begin{array}{*{20}c} u & v \\ \end{array} } \right]^{T} = \left[ {T_{m} \left( {x, y} \right)} \right]\left[ {D_{m} } \right]} & {(m = \, 1, \, 2, \ldots ,n)} \\ \end{array}$$

where [*T*_m_ (*x*, *y*)] is displacement transformation matrix.

In this method, an implicit solution scheme is employed to compute the displacements of element nodes. This is achieved by solving the global equilibrium equation at each time step. Assuming there are ***n*** elements in the model, the equilibrium equation of these elements can be derived based on the principle of minimum potential energy. Specifically, we have:

3 $$\left[ {\begin{array}{*{20}c} {\begin{array}{*{20}c} {K_{11} } & {K_{12} } \\ {K_{21} } & {K_{21} } \\ \end{array} } & \cdots & {\begin{array}{*{20}c} {K_{1n} } \\ {K_{2n} } \\ \end{array} } \\ \vdots & \ddots & \vdots \\ {\begin{array}{*{20}c} {K_{n1} } & {K_{n2} } \\ \end{array} } & \cdots & {K_{nn} } \\ \end{array} } \right]\left[ {\begin{array}{*{20}c} {\begin{array}{*{20}c} {D_{1} } \\ {D_{2} } \\ \end{array} } \\ \vdots \\ {D_{n} } \\ \end{array} } \right] = \left[ {\begin{array}{*{20}c} {\begin{array}{*{20}c} {F_{1} } \\ {F_{2} } \\ \end{array} } \\ \vdots \\ {F_{n} } \\ \end{array} } \right]$$

where *K*_*pq*_ is the 6 × 6 stiffness submatrix; *F*_*p*_ is the force vector; *D*_*p*_ is the deformation variable submatrix of the element *p*, which contains six displacement components, as shown in Eq. (1).

#### (2) Block contact and energy dissipation

The current two-dimensional DDD program focuses on boundary node contact between adjacent blocks, allowing for various behaviors such as opening, closing, sliding, and locking between blocks. There are three primary forms of contacts exist between adjacent blocks, *i.e.*, point-point, point-edge and edge-edge contacts. When the calculation module searches for a contact point, it computes the normal and tangential intrusion distances at that position. Subsequently, it applies normal and shear springs and augments the corresponding stiffness submatrix in the equilibrium equation. Namely, the penalty function method is employed within the software to prevent blocks from overlapping or embedding into each other. Additionally, the program equips blocks with the capability to undergo destruction. For instance, a block composed of multiple elements can fracture into smaller volumes, leading to the formation of new contact surfaces on these fragmented blocks.

For the moving blocks, energy dissipation primarily occurs through friction. When a block contacts with other blocks, sliding may happen along the boundaries of these adjacent blocks, generating the corresponding frictional energy. This process effectively consumes the kinetic energy of the blocks. Assuming that the point *p*_1_ of the block *m* enters the boundary defined by the points *p*_2_ and *p*_3_ of the block *n*, with the entry point *p*_0_ and the normal spring stiffness denoted by *k*_n_, the resulting frictional force can be expressed as follows:

4 $$F = {\text{sgn}} \left( {l_{d} } \right)k_{n} d_{n} \tan \left( {\varphi^{\prime}} \right)$$

where *d*_n_ is the normal intrusion distance and $\varphi^{\prime}$ is the friction angle. The friction force is computed based on the normal contact pressure. *l*_d_ is the effective displacement of *p*_1_ along the  direction compared to *p*_0_, and this displacement helps determine the direction of friction. When *l*_d_ aligns with the direction , it is considered positive, and ${\text{sgn}} (l_{d} ) = 1$. Conversely, when *l*_d_ opposes the direction, it is negative and ${\text{sgn}} (l_{d} ) = 1$.

The vector length of the friction force *F* along the  direction can be calculated by Eq. (5):5

Then, the friction energy generated by the block *m* can be expressed as:

6 $$\prod f = \frac{F}{{\sqrt {\left( {x_{3} - x_{2} } \right)^{2} + \left( {y_{3} - y_{2} } \right)^{2} } }}\left( {u_{1 } v_{1} } \right)\left\{ {\begin{array}{*{20}c} {x_{3} - x_{2} } \\ {y_{3} - y_{2} } \\ \end{array} } \right\}$$

In addition to friction, the dynamic damping is also employed to dissipate the kinetic energy of a block. Assuming that at the end of the current time step, the block has the velocity of *v*. At the beginning of the next time step, the velocity of the block is *v* multiplied by the damping coefficient, and the iteration continues until the dynamic equilibrium is achieved.

#### (3) Damage evolution and strength criteria

To account for the non-uniformity of material properties, the DDD method incorporates the Weibull distribution (Gong et al. 2024) from probability statistics. This distribution can characterize the non-uniformity of rock and soil and the material damage resulting from stress concentration. The relationship can be expressed by Eq. (7):

7 $$\varepsilon = \sigma /E = \sigma /E_{0} \left( {1 - D} \right)$$

where *σ* and *ɛ* are the stress and strain of an element, respectively. *E*_0_ and *E* represent the elastic moduli of the initial and damaged element, and *D* is termed the damage variable. According to the equivalent strain principle, the elastic modulus of an element gradually decreases as damage evolves. The damage variable adheres to both the Mohr–Coulomb and maximum tensile strain criteria, that is:

8 $$f_{c0} \le \sigma_{1} - \frac{1 + \sin \varphi }{{1 - \sin \varphi }}\sigma_{3}$$

9 $$\sigma_{3} \le f_{t0}$$

10 $$D = \left\{ {\begin{array}{*{20}c} {0,} & {\varepsilon _{{t0}} \le \varepsilon < 0} \\ {1 - \frac{{\lambda \varepsilon _{{t0}} }}{\varepsilon },} & {\varepsilon _{{t0}} \le \varepsilon < \varepsilon _{{t0}} } \\ {1,} & {\varepsilon \le \varepsilon _{w} } \\ \end{array} } \right.$$

where *f*_c0_ and *f*_t0_ are the uniaxial compressive and tensile strength, respectively; $\sigma_{1}$ and $\sigma_{3}$ represent the maximum and minimum principal stresses; $\varphi$ is the internal friction angle; $\varepsilon_{tu}$ is the ultimate tensile strain and can be used to characterize whether the element has completely lost its load-bearing capacity; $\varepsilon_{t0}$ represents maximum elastic strain; λ is the residual strength coefficient. When the stress–strain state of an element inner the model satisfies either the Mohr–Coulomb or maximum tensile strain criteria, the element is considered to have experienced either compressive shear or tensile shear failure.

### Numerical configuration and parameter inversion

According to the principles of the DDD method, the velocity attenuation resulting from the collision between blocks and the slope is primarily influenced by the sliding friction angle. Simultaneously, the artificial damping consumes the kinetic energy of blocks at each time step. Therefore, conducting rockfall hazard analysis necessitates determining both the sliding friction angle and the damping coefficient. Prior to this analysis, typical blocks are selected on the basis of the effect of falling-rock shape and size on trajectory. Subsequently, the parameters affecting rockfall movement such as the initial position of the blocks, the height of protective structures, cohesion and elastic modulus are determined. Finally, a two-dimensional numerical model is established for parameter inversion and subsequent rockfall analysis.

#### (1) Model establishment

**Fig. 6:**
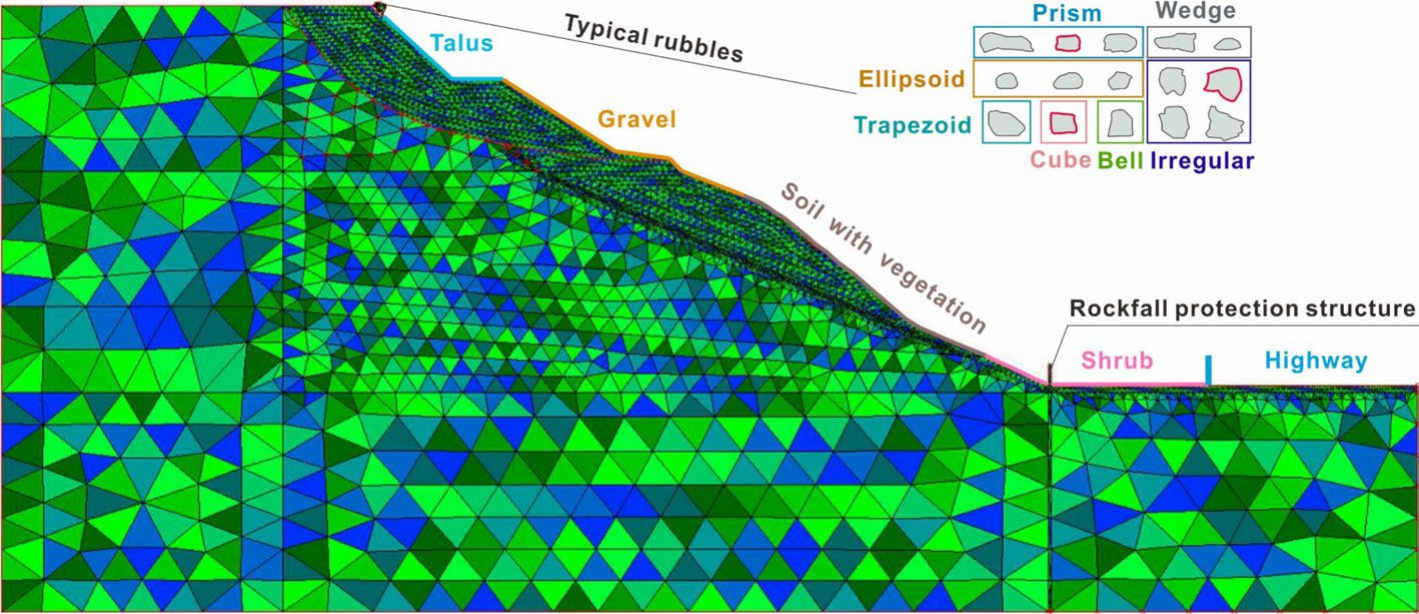
Numerical model for individual rockfall

Based on the distribution of rubble, 15 typical blocks with 7 different shapes are selected to establish rockfall models, as depicted in Fig. 6. To conservatively assess the risk of rockfall, the initial block is positioned at the slope crest during the parameter inversion and rockfall analysis, considering the various positions of each block within the source area. The slope properties are assigned based on the material distribution. Specifically, the geomaterials include talus, gravel, soil with vegetation, and shrub from top to bottom. Simultaneously, the simulation does not account for slope deformation. The model employs the vertical sliding supports on both sides and fixed supports at the bottom boundary. In the rockfall analysis, a 3 m × 0.5 m rockfall protection structure is placed at the slope toe, with the lower part embedded in the bedrock to a depth of 1 m. Note that the protection structure is unnecessary for the parameter inversion. Additionally, the slope mesh around the free surface is locally densified, and the slope material is characterized by high shear strength and elastic modulus to maintain stability. The parameters of slope materials are listed in Table 1.

**Table 1:** Physical and mechanical parameters of the slope materials

Slope material	Weight (kN/m^3^)	Elastic modulus (kPa)	Poisson's ratio	Cohesion (kPa)	Tensile strength (kPa)
Talus	19	4.6 × 10^6^	0.23	9 × 10^5^	5 × 10^4^
Gravel	20.5	4.8 × 10^6^	0.25	9.1 × 10^5^	5.2 × 10^4^
Soil with vegetation	22	5 × 10^6^	0.28	9.3 × 10^5^	5.4 × 10^4^
Shrub	23	5.3 × 10^6^	0.31	9.5 × 10^5^	5.6 × 10^4^
Rockfall block	26	2 × 10^7^	0.35	8 × 10^6^	8.25 × 10^5^
Protection structure	20	6 × 10^6^	0.3	–	2 × 10^5^

#### (2) Parameter inversion

**Fig. 7:**
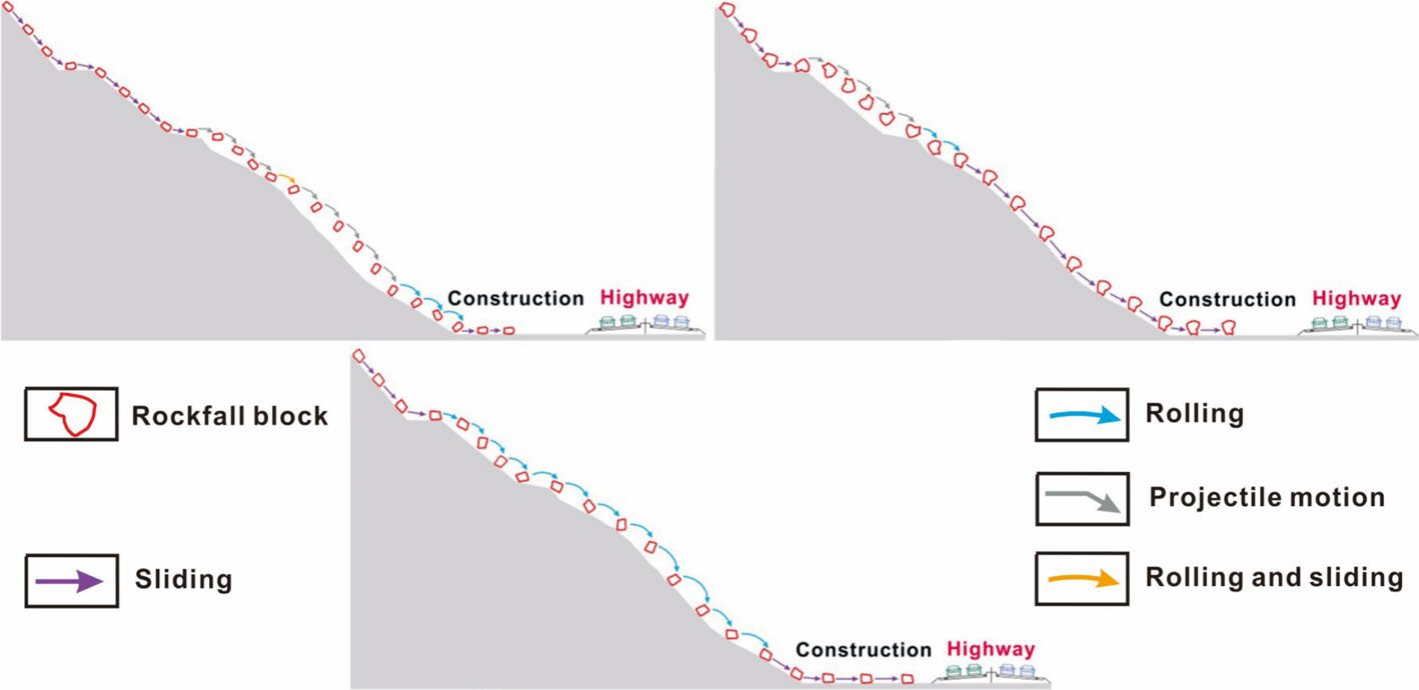
Rockfall trajectories obtained by the parameter inversion (Note: the falling rocks are magnified 1.7 times for clear observation)

In the DDD method, the damping coefficient is applied to the entire model, whereas the friction angle serves as a parameter specific to each contact surface. Essentially, the simulation requires the inversion of five parameters. Given the complexity of multiple inversion parameters, one approach involves increasing the damping coefficient and collectively reduce the friction angle across all slope surfaces can be employed. Firstly, based on the recovery coefficients from the collision test (Guzzetti et al. 2003; Giani 1992; Hungr 1988), the ratio between the recovery coefficients of four slope surfaces is calculated. According to this ratio, the initial friction angles for the slope surface materials from top to bottom are set as 10°, 8.5°, 11.6°, and 6.7°. Subsequently, based on the relationship between damping and recovery coefficients (Yan et al. 2022; Zhu et al. 2017), the initial damping coefficient value is set as 0.88. Finally, three typical types of rocks are selected from the rockfall accumulation area at the slope base, *i.e.*, prismatic, cubic, and irregular blocks, varying in size from small to large as highlighted in red in Fig. 7. The cross-section and shape of these rocks are incorporated into the numerical simulation. When the simulated rockfall positions cannot match the actual monitoring results, the friction angle and restitution coefficient are adjusted simultaneously until the consistency is achieved.

### Individual rockfall

The numerical results for individual rockfalls indicate that none of the 15 blocks managed to leap over the protection structure. However, one wedge, two irregular blocks, and a small prism caused the significant deformation of the protective structure with a maximum value exceeding 0.05 m, and some of them even result in structural damage. The spherical rubble rolled along the slope until it came to rest in the shrub area without making contact with the structure, indicating a pattern consistent with the observed rockfall accumulation at the slope toe, as shown in Fig. 5b. The medium and large prism blocks slid along the slope and eventually halted at the first and second platforms, respectively. As for the remaining rubble, despite colliding with the structure, they did not cause any significant deformation. The following will delve into the motion characteristics and dynamic behaviors of the four blocks responsible for the structural deformation. For ease of reference, the two irregular blocks will be named Block 1 and Block 2, respectively. The masses of these four blocks follow the order: Block 1, Block 2, wedge, and prism from largest to smallest.

**Fig. 8:**
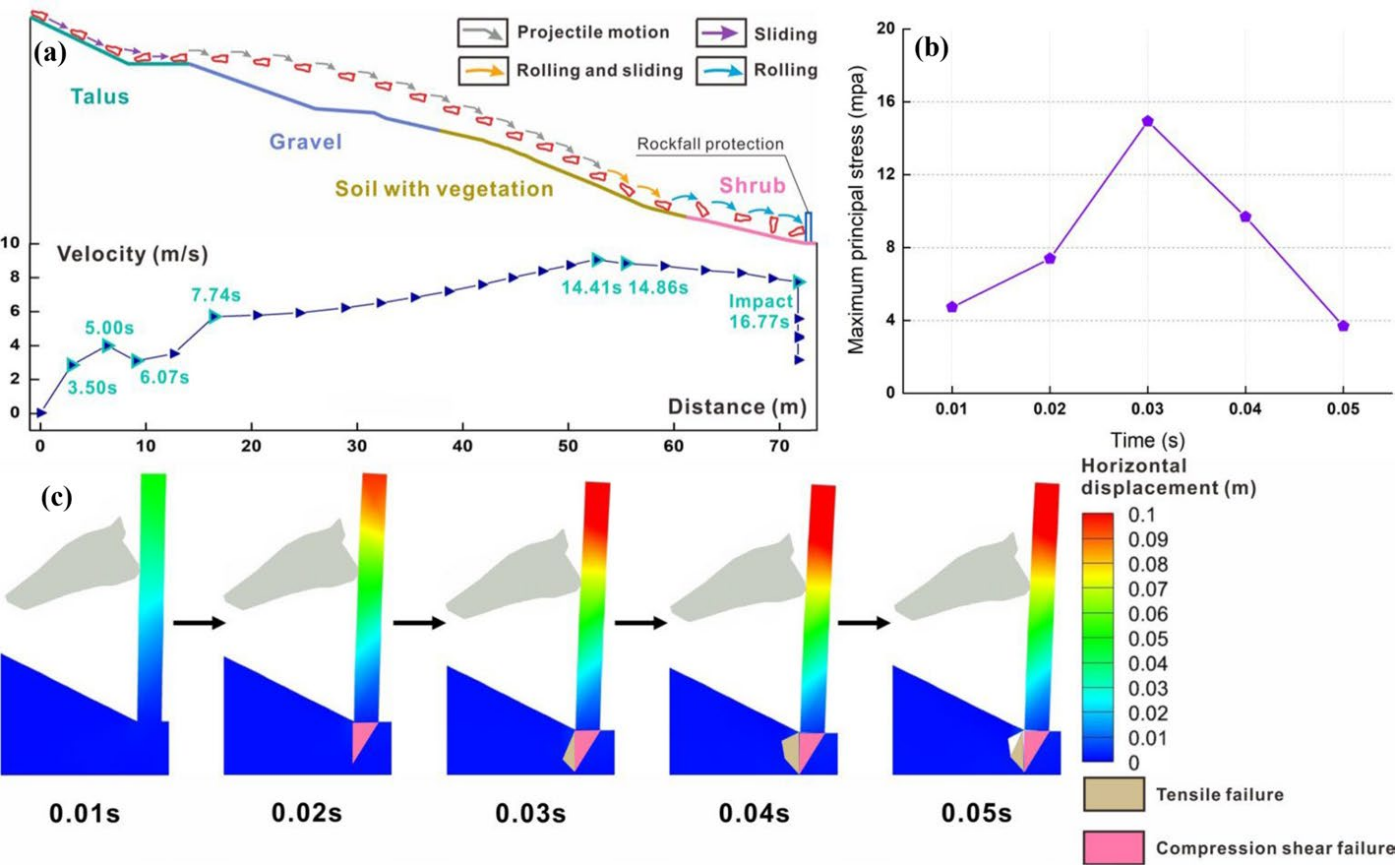
Numerical results of wedge block: **a** trajectory with the 1.7 times magnified block and protective structure, and linear velocity-distance curve; **b** maxiumum principal stress and **c** horizontal displacement of protection structure under impact

Figure 8a depicts the velocity-distance curve and trajectory of the wedge. Each data point in the figure precisely aligns with the position of the block directly above it. To clearly illustrate the block posture, the slope is graded, and the block is magnified 1.7 times. Figure 8a reveals that the wedge-shaped block undergoes sliding and rolling during the initial and final stages, as well as horizontal projectile motion in the intermediate stage. In the initial stage, owing to the low speed of the block, the influence of friction and the collision with the platform, the trajectory closely follows the slope surface. At 6.07 s, the speed increases to 3.52 m/s, marking the onset of large horizontal projectile motion. In the middle stage, the small windward area of the wedge leads to a significant speed increase after it detaches from the slope surface. At 14.86 s, the block collides with the vegetation surface, reaching a peak speed of 9.06 m/s just 0.45 s before the collision. In the final stage, although the block is unlikely rotate due to its shape, it briefly slides along the slope before fully transitioning into rolling at high speed. Eventually, it collides with the middle and upper part of the structure with a speed of 7.85 m/s at that moment.

Although the wedge rank only third among the four types of blocks in terms of quality, it can cause bending and tipping damage to protection structures at a high speed. The collision duration is a mere 0.05 s. In this analysis, the structure is considered as a cantilever beam rotating 90°, with a concentrated force acting on the upper middle part near its free end. The exposed section of the structure bears interlayer shear force, while the root embedded in the rock and soil mass experiences the maximum bending moment. When the block first contacts the structure, the average horizontal deformations at the upper and middle parts are 0.05 m and 0.02 m, respectively. The maximum principal stress at the impact point of the structure is 4.73 MPa, as depicted in Fig. 8b. As the collision persists, both displacement and stress continue to increase. The structure undergoes bending deformation akin to that of a cantilever beam, with displacement gradually decreasing from the end downwards. The compression shear failure occurs at the root, as illustrated in Fig. 8c. At 0.03 s, the bending deformation reaches its maximum, and the bending moment borne by the root attains its peak anti-overturning moment. Tensile failure begins to occur on the impacted side. During this phase, the upper structure remains connected to the lower part, and the maximum principal stress reaches 14.93 MPa. By 0.04 s, the upper structure starts to fracture along the root, transitioning from bending deformation to overturning failure. The tensile failure of root further develops. Eventually, due to the loss of the structural resistance, the maximum principal stress begins to decrease, reaching its lowest point at 0.05 s, resulting in complete fracture of the upper structure along the root. As a result, the protective structure is destroyed, allowing the block to enter the renovation area and threaten the safety of construction workers and equipment.

**Fig. 9:**
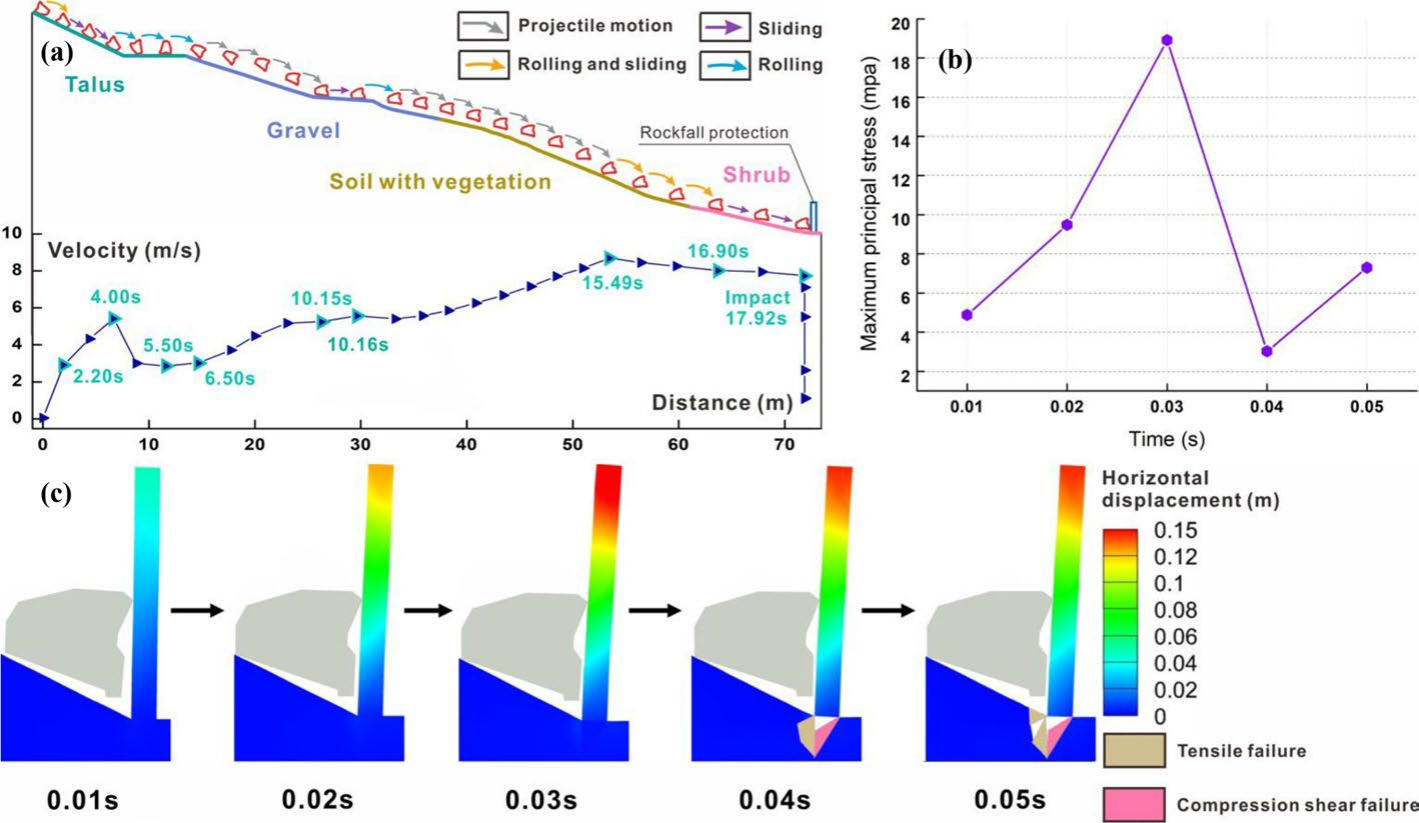
Numerical results of Block 1: **a** trajectory with the 1.7 times magnified block and protective structure, and linear velocity-distance curve; **b** maximum principal stress and **c** horizontal displacement of protection structure under impact

Figure 9a illustrates the velocity-distance curve and trajectory of Block 1. During the initial and final stages, the block exhibits sliding, while in the middle stage, it primarily engages in flat throwing motion. During the start-up phase, the block slides down the talus surface and reaches the toe in 4 s with a linear velocity of 5.42 m/s. It then collides with the secondary platform, resulting in a decrease in velocity and a transition from sliding to rolling. At 6.5 s, the block speed increases to 3.03 m/s, initiating horizontal projectile motion. However, due to its substantial weight and volume, this phase lasts only 3.65 s before colliding with the first-level platform. After a short sliding along the platform, the block enters its second projectile motion. It starts with a velocity of 5.57 m/s, which exceeds the initial velocity of the first flat throwing motion. Consequently, both the duration and distance of this movement are extended. At 15.49 s, the block encounters vegetation, reaching a peak velocity of 8.69 m/s. After stabilizing its posture, it continues to slide along the slope and eventually collides with the lower part of the structure at 17.92 s with the velocity of 7.71 m/s.

The total collision time for Block 1 with the protection structure is 0.05 s. Due to the substantial speed and mass of the block, the impact topples the structure. When the block contacts the middle and lower parts of the structure, the maximum principal stress at the impact point reaches 4.88 MPa, as depicted in Fig. 9b. Meanwhile, the upper and middle parts of the structure exhibit average horizontal displacements of 0.05 m and 0.03 m, respectively, as shown in Fig. 9c. By 0.02 s, the displacement increases significantly with the values at the end, upper middle, and lower parts reaching 0.11 m, 0.09 m, and 0.05 m, respectively. This deformation is essentially equivalent to what occurs when the structure reaches its peak stress under the impact of the wedge. Notably, no compressive shear or tensile failure occurs at the root, indicating that the superstructure remains relatively unbent. At 0.03 s, both stress and deformation reach their peaks. The former reaches 18.91 MPa, the end displacement of the latter reaches 0.15 m, and the root remains undamaged. However, by 0.04 s, the superstructure fractures completely along the root. Compression shear and tensile damage occur at the root, causing the stress to sharply drop to 3.02 MPa, ultimately resulting in the structure toppling. The substantial block mass renders the structure utterly incapable of resisting the impact of rockfall, leading to its collapse under the rapid increase in speed. As the collision concludes, the damaged upper structure rotates in the tilting direction, inducing tensile failure in the adjacent soil at the root. This process also lets the structure compresses the falling rock, causing the maximum principal stress to rise to 7.29 MPa. Considering the large volume and high impact speed of the block, its entry into the renovation area may cause casualties and severe damage to construction equipment.

**Fig. 10:**
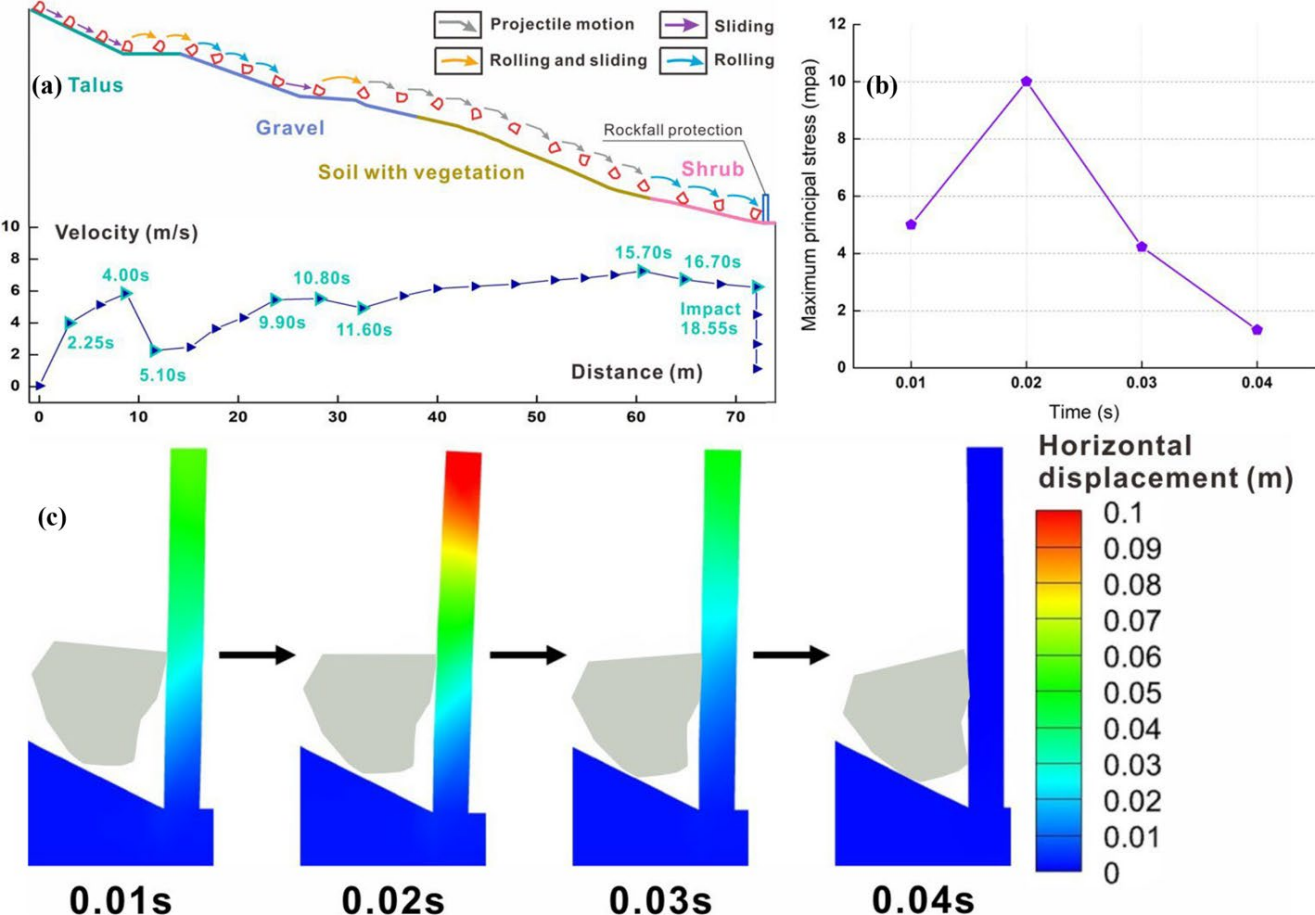
Numerical results of Block 2: **a** trajectory with the 1.7 times magnified block and protective structure, and linear velocity-distance curve; **b** maxiumum principal stress and **c** horizontal displacement of protection structure under impact

Figure 10a depicts the velocity-distance curve and trajectory of Block 2. Throughout its motion, the block maintains rotation except the initial stages when it slides along the slope. The primary modes of motion are rolling and flat throwing. During the start-up phase, the block slides along the third-level slope and subsequently collides with the platform, transitioning to rolling motion along the slope. At 9.9 s, the block reaches the foot of the second-level slope, with its speed increasing to 5.45 m/s. Then, it begins a flat throwing motion, and its trajectory moves away from the slope. Particularly, the block speed increases gradually during this process due to continuous rotation, which results in significant air resistance. At 15.7 s, the block jumps over the vegetation surface, achieving the peak velocity of 7.25 m/s. After a mere 0.85 s, it collides with the shrub surface and starts rolling, gradually decreasing in speed. Finally, the block collides with the lower part of the protection structure with the speed of 6.23 m/s.

The collision between Block 2 and the protection structure lasts for 0.04 s and do not result in any structural damage. Due to the rotation of the block during its collision with the structure, the contact type changes from the point contact to the surface contact, and the data at the initial collision point is extracted to plot the maximum principal stress-time curve. At 0.01 s, the front tip of the block contacts with the middle and lower parts of the structure, generating a maximum principal stress of 5.01 MPa at the impact point, as depicted in Fig. 10b. The average horizontal deformation from the end to the middle of the structure is 0.04 m, while the deformation at the middle and lower parts is 0.03 m, as illustrated in Fig. 10c. As the collision continues, both the stress and structural displacement at the impact point further increase. The stress reaches 10 MPa, and the average values at the end, middle, upper, and lower parts of the structure increase to 0.095 m, 0.065 m, and 0.036 m, respectively. Comparing the dynamic response characteristics of the structure at 0.02 s with the features during the corresponding time of the structure under Block 1 impact, the displacements and impact forces of the structure remain essentially similar. Since the mass of Block 2 is only slightly smaller than that of Block 1, if the former continues to impact the structure along the tip, it could potentially cause structural toppling. However, at 0.03 s, the block rotates, transitioning from the sharp endpoint impact to the impact on the entire protruding surface. This rotation significantly reduces the impact force of the block, resulting in a maximum principal stress at the initial impact point of 4.22 MPa, which is lower than the value when the block directly contacts the structure.

**Fig. 11:**
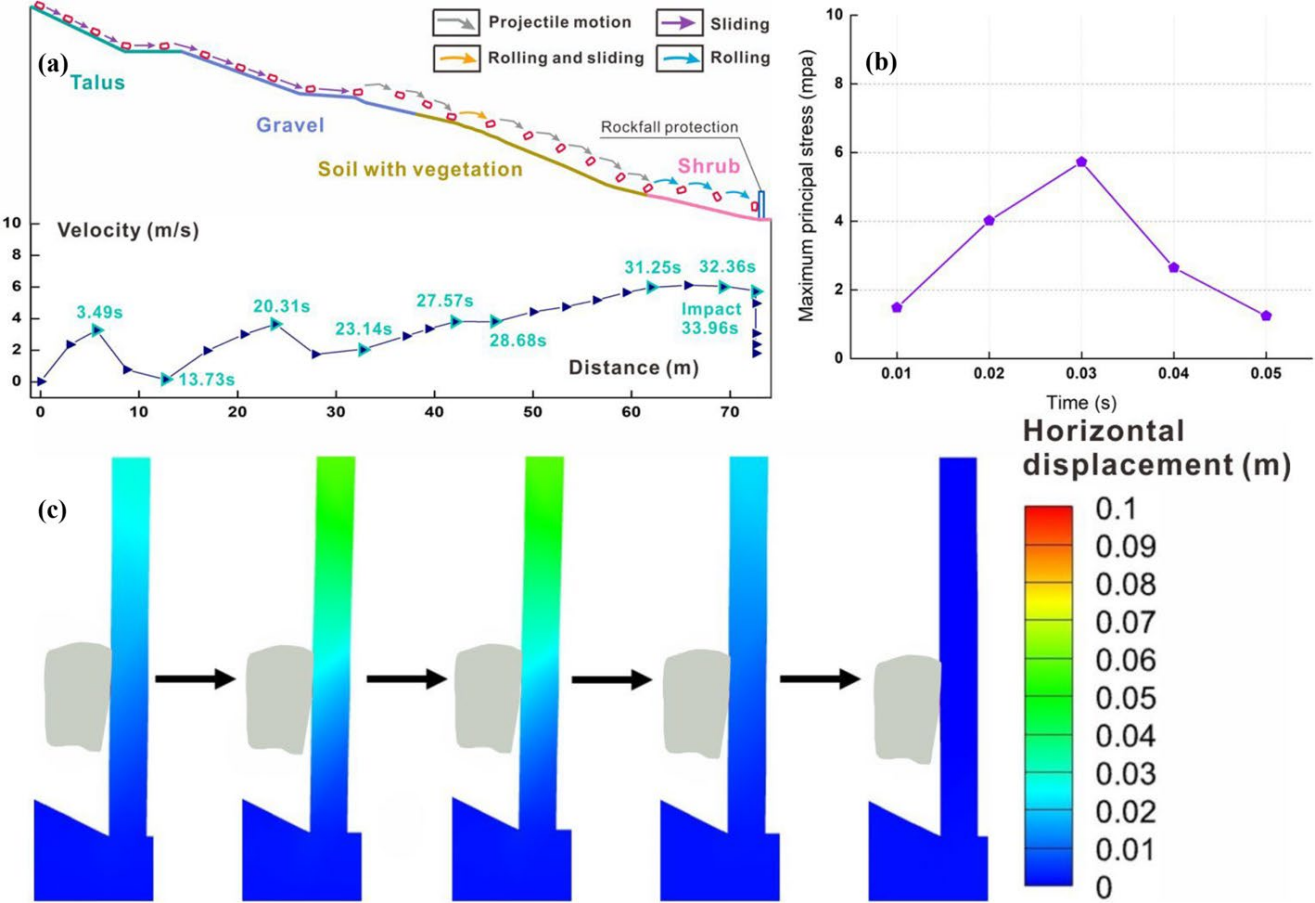
Numerical results of prism block: **a** trajectory with the 1.7 times magnified block and protective structure, and linear velocity-distance curve; **b** maxiumum principal stress and **c** horizontal displacement of protection structure under impact

Figure 11a illustrates the velocity-distance curve and trajectory of a prism. Throughout the entire movement process, the block initially slides along the talus and gravel surface, executes two flat throwing movements on the first-level slope covered with vegetation, and ultimately rolls on the shrub slope until it collides with the protection structure. In the early stage of movement, the block continues sliding along the slope surface. However, due to the frictional force on the slope and multiple collisions with the platform, the velocity of the block is difficult to increase. At 23.14 s, the speed is a mere 2.07 m/s. In the mid-term stage, the block begins to perform a flat throwing motion, but its lower speed causes a brief collision with the slope surface. Finally, in the last stage, the block contacts the shrub surface, achieving the peak velocity of 6.11 m/s. As it rolls along the slope, it gradually decelerates until it collides with the structure, and the velocity decreases to 5.76 m/s.

Due to the low mass and impact velocity, as well as its collision with the structure, the block does not cause significant deformation or damage to the structure. The collision between the block and the structure lasts for a total of 0.05 s. Because the upper surface of the block is in contact with the protection structure from the beginning of collision, the data in the maximum principal stress-time curve represent the average stress values on the contact surface. Throughout the entire collision process, the displacement of the block and the stress at the impact point increased with time, reaching the peak at 0.03 s with the maximum principal stress of 5.72 MPa. The average displacement from the end to the middle of the upper structure is 0.055 m, while the displacement from the middle and lower parts is 0.03 m. Simultaneously, the block velocity decreases to 3.06 m/s. Later, the structural displacement gradually decreases, and the deformation fully recovers at 0.05 s.

The simulation results for the four blocks reveal that the falling rocks exhibit a flat throwing motion above the vegetation-covered slope, with limited continuous contact with the surface. Consequently, constraining their velocity becomes high. Especially, the deformation and damage resulting from the impact of the falling-rock tip on the structure far exceed those caused by the surface impact. This phenomenon aligns with the findings from both the experiment (Wang et al. 2022) and numerical simulation (Yu et al. 2021b). Furthermore, when the falling rock makes contact with the structure, the stress generated by the impact is not maximal initially but increases to a peak as deformation progresses. This behavior is consistent with the impact force–time curve observed in the studies involving falling rock impacting sand cushion layers (Shen et al. 2019), flexible protective nets (Tian et al. 2023), and bridges (Yuen et al. 2023). Notably, when the structure experiences damage or rebounds, the maximum principal stress decreases. In summary, two of 15 blocks can cause structural damage, resulting in a failure probability of 13.3%. This suggests that the structure is ineffective in protecting against the individual rockfall disasters.

## Analysis of cluster rockfall

### Slope stability analysis

Based on the survey and monitoring results, it has been observed that the quarry fill could become unstable during rainfall, potentially leading to the cluster rockfall hazard. To address this problem, the slope stability analysis is conducted in this Section based on the slope materials obtained from geological exploration, as shown in Fig. 3a. Firstly, the meteorological data and specification from the Hydrology Bureau of the Ministry of Water Resources of the People’s Republic of China (2009) are consulted. Subsequently, the Midas finite element software is employed to analyze slope stability under the influence of Typhoon Capricorn, Typhoon Rumbia, and rainstorm occurring once in 10 years. These three distinct rainfall conditions persisted for 3 days with the cumulative rainfalls of 58.7 mm, 175.5 mm, and 322 mm, respectively. When the quarry fill reaches an unstable state under specific conditions, the failure surface can be identified from the simulated results and incorporated into the DDD model as the sliding surface for subsequent dynamic analysis.

The rainfall simulation employs the transient analysis module, and the Van Genuchten model is utilized to calculate the soil permeability coefficient based on the seepage test results (Zhao 2016). Given that the limestone layer has a low permeability coefficient and lies deep within the slope, its permeability characteristics are not considered into the analysis. The physical and mechanical parameters are determined based on the geological survey data, and the specific values for the rock and soil mass are listed in Table 2.

**Table 2:** Parameters of rock and soil

Types of rock and soil layers	Elastic modulus (kPa)	Weight (kN/m^3^)		Cohesion C (kPa)	Internal friction angle φ (°)	V-G model parameters				
		Natural	Saturated			*a* (kPa)	*n*	*m*	$\theta_{r}$	$\theta_{s}$
Quarrylandfill	1.2 × 10^5^	18.5	20	12	10	10	1.56	0.3589	0.02	0.23
Silty clay	3.4 × 10^5^	20	21.7	9	31	10	1.9	0.4736	0.04	0.28
Moderately weathered limestone	5.8 × 10^6^	23.5	23.7	450	33	–	–			

**Fig. 12:**
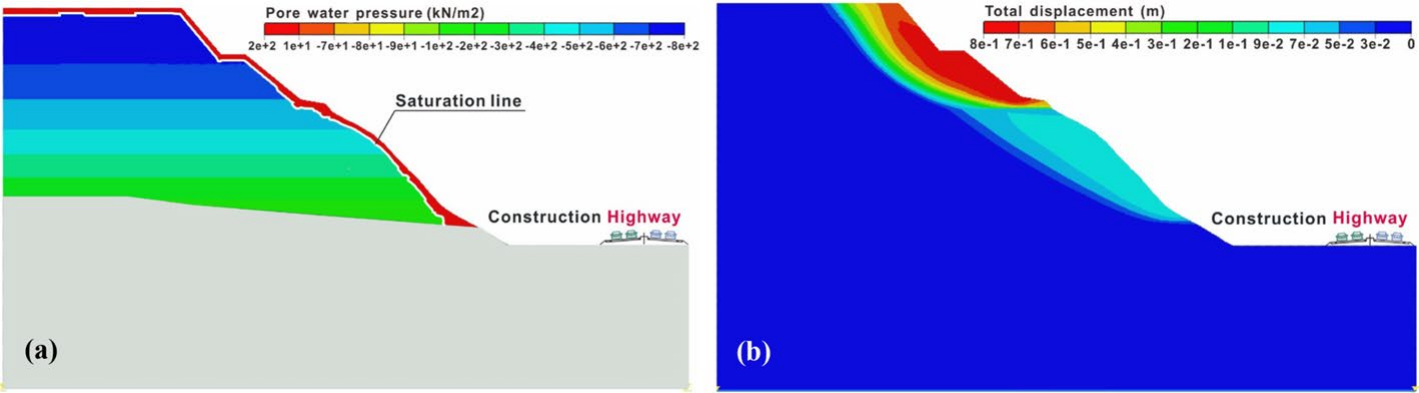
Seepage and deformation characteristics of slope under rainstorm occurring once in 10 years: **a** pore water pressure; **b** total displacement

The findings reveal that as rainfall increases, the factor of safety (FoS) gradually decreases. Specifically, the FoSs of the slope under Typhoon Capricorn, Typhoon Rumbia, and rainstorm occurring once in 10 years are 1.018, 1.013, and 0.977, respectively. During the rainstorm, the slope experiences shallow saturation, leading to the landslide of the quarry landfill, as shown in Fig. 12a and b. The subsequent analysis of cluster rockfall will characterize the unstable soil based on the sliding surface. Under the Typhoon condition, the safety factor of the slope falls below 1.05, indicating a less stable state according to the design code (Ministry of Transport of the People's Republic of China 2015). This observation aligns with the on-site monitoring, which shows that cracking and deformation of the slope intensify after rainfall.

### Cluster rockfall

According to the sliding surface in Fig. 12b, the slope instability area is imported into the DDD software. The 15 typical blocks are stacked on the third-level slope to simulate the cluster rockfall. Because of the landslide movement and mesh discretization, the material tensile strength and cohesion are assumed as 1.7 MPa and 0.5 kPa, respectively. Additionally, recognizing that some of the shallow landfill bodies are saturated when the slope becomes unstable, a weight of 19.3 kN/m^3^ is assigned to the sliding body material. The remaining parameters of the landslide align with those specified in Table 1, and the slope material corresponds to that of the individual rockfall model.

**Fig. 13:**
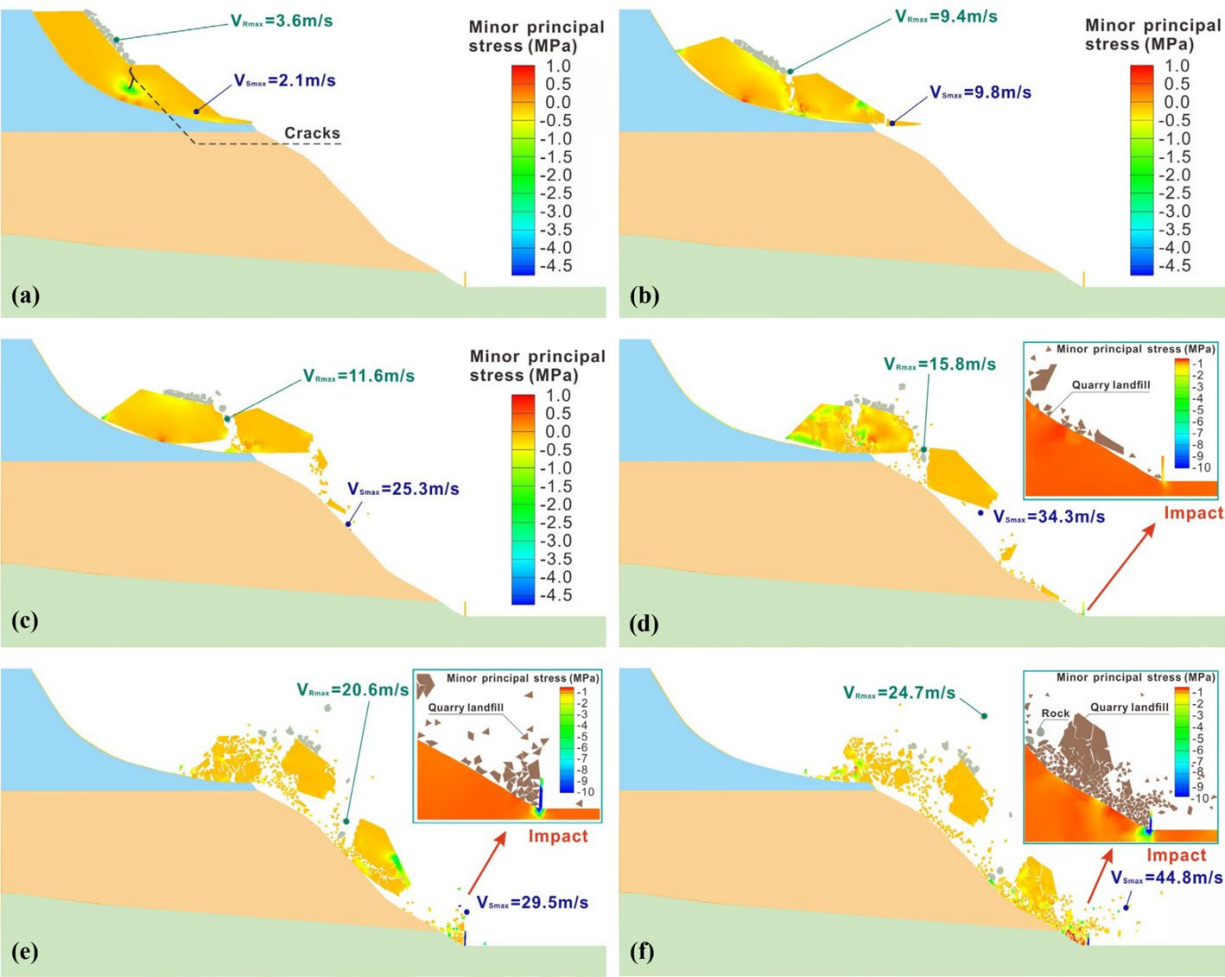
Simulated movement of cluster rockfall at different time: **a** 0.45 s; **b** 2.15 s; **c** 3.35 s; **d** 4.63 s; **e** 5.26 s; **f** 5.76 s

Figure 13 illustrates the process of landslide and rubble movement after the slope instability. Note that when compared to the individual rockfall events, the cluster rockfall induced by the landslide exhibits significantly greater movement speed and impact force. Initially, the sliding body descends gradually along the fracture surface. The soil movement speed below the secondary platform surpasses that of the upper part, reaching a maximum value of 2.1 m/s. This speed results in stress concentration within the middle of the sliding body, leading to the formation of a crack, as shown in Fig. 13a. Simultaneously, the accumulated rubble on the slope begins to move due to the inertia. The central block within the rubble experiences a higher movement speed (approximately 3.6 m/s) than the overall landslide. This difference indicates the relative motion between the block and the landslide body.

At 2.15 s, the velocity of the sliding body significantly increases, resulting in the complete connection of the middle crack to the upper surface. This connection occurs due to the amplified velocity difference between the sliding bodies on both sides. Concurrently, the soil at the shear outlet detaches from the slope and is also separated from the sliding mass, reaching a maximum velocity of 9.8 m/s, as depicted in Fig. 13b. Affected by crack expansion, the rubble loses its effective mechanical support and begins to fall into the crack with a maximum speed of 9.4 m/s. By 3.35 s, the entire landslide body is broken along the crack. The high velocity of the sliding body at the front of the crack causes the rear part to tilt backward due to the inertia after losing traction from the front. Remarkably, it even bounces off a surface block, as shown in Fig. 13c. Simultaneously, the landslide mass in front of the crack starts to disintegrate, with loose gravel soil separating from the main landslide mass under its own weight and gradually fragmenting.

At 4.63 s, a small portion of the soil migrates to the slope toe and collides with the protective structure. Meanwhile, the rear sliding body begins to disintegrate and rebound off a rough stone on the slope. Notably, the speed of the block that has previously fallen along the crack further increases and reaches 15.8 m/s. At 5.26 s, the rear landslide has completely disintegrated and ejected a substantial amount of rubble to the air. This rubble begins to move obliquely, culminating in a mass rockfall disaster. The protective structure at the slope base experiences significant deformation due to the continuous impact of the landslide mass. Analyzing the stress contour reveals a pronounced stress concentration phenomenon within the protective structure, particularly from the end to the middle and upper parts. As the landslide mass accumulates along the foot of the slope, the protective structure bears significant static loads, as indicated in Fig. 15e. In the final stage, the front main sliding mass and two falling rocks reach the slope base, causing a complete fracture of the protective structure along its foundation. Some of the sliding masses even cross the protective structure, infiltrating the construction area. Simultaneously, the speed of falling rocks in the rear group surges to a maximum value of 24.7 m/s. At this velocity, the landslide is likely to carry rubble that could cover the entire construction area, leading to severe equipment damage and even casualties.

Overall, the mechanism underlying landslide-induced mass rockfall disasters is inherently intricate. Initially, the accumulation of rubble on the third-level slope acts as a load, slowing down the movement of the rear landslide mass. This deceleration results in a velocity difference between the landslide mass on both sides along the foot of the slope, and the differential velocities lead to a fracture along the middle of the landslide mass. Subsequently, the fracture causes the rear sliding body to tilt backward, effectively bouncing away the accumulated blocks on the slope. As the rear landslide body breaks and collapses along its own middle, it continues to collide with blocks. This collision results in the ejection of a large amount of rubble, which begins to move obliquely, forming cluster rockfall disasters. Ultimately, due to the loose structure of the landslide body itself, it continuously disintegrates and breaks during movement. This process gives rise to multiple micro landslide bodies with high kinetic energy. The cumulative impact of dynamic and static loads causes the protective structure to fail.

From the protection perspective, the structure proves inadequate in withstanding the impact of the sliding body. Furthermore, it faces considerable challenges in preventing both the sliding body and the group of falling rocks from breaching its boundaries and entering construction and road areas. After analyzing the results of rockfall events, it can be found that this protective structure can only effectively bear the impact of small to medium rubble. However, when confronted with large blocks or soil-rock mixture disasters triggered by landslides, the protective structure becomes ineffective. Consequently, it may have significant risk to utilize this kind of protective structures on a slope consisting of filling materials.

## Cost–benefit analysis of protection structure

In terms of the rockfall protective effect, the combined structure of bamboo springboards and steel pipes is unable to intercept the cluster rockfall and two of the 15 classical individual rock blocks. Undoubtedly, adopting this protective facility will bring high risks to the personnel and machinery at the slope bottom during the construction. However, considering the relatively low cost of the protective structure, it is necessary to analyze its cost-effectiveness and propose the improved disaster mitigation strategies. In this section, the economic loss and the probability of casualties caused by falling rocks with and without protection are estimated using Eq. (11) according to the literature (Hungr et al. 2005):

11 $$P_{{\text{LOL(PROP)}}} = P_{{\text{L}}} \times P_{{\text{T:L}}} \times P_{{\text{S:T}}} \times V \times E$$

where *P*_*LOL*_ and *P*_*PROP*_ refer to the annual probability of casualties and economic loss caused by rockfall, respectively. *P*_*L*_ is the annual probability of rockfall occurrence. *P*_*T:L*_ is the probability of rockfall reaching the personnel and machinery. *P*_*S:T*_ is the spatiotemporal probability of personnel and machinery appearing in the rockfall impact area. *V* is the vulnerability. *E* is the economic value, and for the personnel, this value is 1 because the calculation is regarding the probability of casualties.

Based on the on-site observation, individual rockfalls occur almost every month, which means the annual probability of rockfall without protection is 100%. With protection, according to the interception rate of the structure against 15 types of individual rockfalls, the annual probability of the disaster is $\frac{2}{15}$≈13.3%. Meanwhile, since the protective structure cannot intercept cluster rockfalls, the probability of disaster occurrence is therefore controlled by the triggering event, which is the 10% probability of the rainstorm occurring once in 10 years. The *P*_*T:L*_ can be calculated by the ratio of the rockfall impact area to the construction zone. The *P*_*S:T*_ is equal to the ratio of the daily working hours to the total time (24 h). The other parameters are estimated based on the impact force of the rockfall and the economic value of machinery. The parameters for analyzing the potential risk of rockfall hazards are shown in Table 3.

**Table 3:** The parameters for rockfall risk assessment

Type of rockfall	protection	Rockfall-bearing body	*P*_*L*_	*P*_*T:L*_	*P*_*S:T*_	*V*	*E*
Individual	None	People	1	0.4	0.333	1	1
	Yes		0.133				
Cluster	None		0.1	1			
	Yes						
Individual	None	Equipment	1	0.4		0.5	2.33 million RMB
	Yes		0.133				
Cluster	None		0.1	1		1	
	Yes						

After calculation, the economic loss caused by individual and cluster rockfalls without protection are 155,100 RMB and 77,220 RMB, while the probabilities of casualties are 13.2% and 3.33%, respectively. With the protection being applied, the economic loss and the probability of casualties caused by individual rockfalls are reduced to 20,600 RMB and 1.77%, respectively. The protective structure significantly reduces the risk of individual rockfalls. Meanwhile, considering that its direct cost is only around 20,000 RMB, the cost-effectiveness of the protective structure is relatively high. However, it is worth noting that the protective facility cannot effectively prevent the possible casualties resulted from cluster rockfalls.

Due to the infeasibility of closing the existing expressway and given that the uncontrolled rockfalls may move into the road area, the active prevention measures for removing the hazardous rock mass or the carrier should be adopted. Meanwhile, the passive measures are still required to intercept individual or cluster rockfalls. Moreover, the numerical results indicate that the semi-rigid protective facilities for intercepting falling rocks is inappropriate, as these structures are likely to become unstable and lose their protective capability completely after impact. Consequently, it is suggested that a multi-level protective measure combining flexible and rigid protective structures should be applied. The inner passive protection nets can be installed near the toe of the slope to intercept large rock masses, and the outer steel profiles combined with concrete slabs can be placed near the toe of the slope to withstand the impact of smaller rock masses and the sliding body.

## Conclusion

1. In view of the risk of individual rockfall and landslide-induced cluster rockfall disasters existing on the quarry landfill slope, the DDD method is employed to analyze the motion characteristics of single irregular falling rocks and the dynamic response of the protective structure under impact. Meanwhile, this method is integrated with the finite element method to investigate the entire process of the slope instability and subsequent cluster rockfall disaster, as well as the movement characteristics of soil-rock mixtures and their implication on the protective structure. Besides, the quantitative assessment of the consequences of rockfall hazards and the cost-effectiveness of protective structures is conducted, and the prevention measures for rockfalls are therefore proposed. The results show that the structure can effectively withstand the independent impact of small and medium-sized rubble. However, when faced with large blocks or soil-rock mixture disasters resulting from landslides, the protective structure becomes completely ineffective. Although the current protective structures are relatively cost-effective, the extremely high probability of casualties makes them inappropriate.
2. For the semi rigid protection structure partially embedded in the soil, the geometry and mass of individual falling rocks, the impact position on the protection structure and the impact area have a significant influence on its dynamic response because of rockfall. When a wedge-shaped rock impacts the upper part of the structure, it tends to undergo bending tilting failure. Namely, the compression shear fracture occurs at the end of the structure. Conversely, when the irregular blocks with large volumes impact its lower part, the structure often experiences direct toppling failure. Namely, the structure fractures directly along the end without compression or shear failure as a precursor, and the upper structure remains unbent. In addition, the impact of falling rocks causes deformation and damage far exceeding that caused by plane impact. The stress generated by the contact with the structure is not at its maximum. Actually, it gradually increases to a peak as deformation occurs. When the structure is damaged or rebounds, the impact stress significantly diminishes.
3. For the slope consisting of filling masses, the interaction between filling materials and accumulated rough stones on its surface significantly amplifies the risk and complexity of rockfall disasters. On the one hand, the loose filling mass lacks the effective bearing capacity for protective structures and limits the rational arrangement of protective structures. On the other hand, the instability of the filling body, for example caused by rainfall, may lead to the loss of effective support for blocks and trigger cluster rockfall. Furthermore, during the mass movement, the accumulated blocks on the slope slow down the sliding of some filling materials. The differential velocities before and after the landslide can cause fractures at the middle of the slope. Consequently, the rear sliding body tilts backward, leading to the ejection of a substantial amount of rubble and initiating the oblique throwing motion, and evenly the massive rockfall disaster forms.

## Data Availability

The data underpinning this publication can be accessed from Brunel University London's data repository, Brunelfigshare, under a CCBY licence with the DOI of 10.17633/rd.brunel.27652422.
